# Psychometric characteristics of the short form 36 health survey and functional assessment of chronic illness Therapy-Fatigue subscale for patients with ankylosing spondylitis

**DOI:** 10.1186/1477-7525-9-36

**Published:** 2011-05-22

**Authors:** Dennis A Revicki, Anne M Rentz, Michelle P Luo, Robert L Wong

**Affiliations:** 1 Outcomes Research, United BioSource Corporation, Bethesda, MD, USA; 2 Formerly Abbott Laboratories, Global Health Economics & Outcomes Research, Abbott Park, IL, USA; 3 Abbott Laboratories, Abbott Immunology, Parsippany, NJ, USA

**Keywords:** Ankylosing spondylitis, Health-related quality of life, Physical and mental health, Reliability, Validity

## Abstract

**Background:**

We evaluated the psychometric characteristics of the Short Form 36 (SF-36) Health Survey and the Functional Assessment of Chronic Illness Therapy (FACIT)-Fatigue subscale in patients with ankylosing spondylitis (AS).

**Methods:**

We analyzed clinical and patient-reported outcome (PRO) data collected during 12-week, double-blind, placebo-controlled periods of two randomized controlled trials comparing adalimumab and placebo for the treatment of active AS. The Bath Ankylosing Spondylitis Disease Activity Index, Bath Ankylosing Spondylitis Functional Index, and other clinical measures were collected during the clinical trial. We evaluated internal consistency/reliability, construct validity, and responsiveness to change for the SF-36 and FACIT-Fatigue.

**Results:**

The SF-36 (Cronbach alpha, 0.74-0.92) and FACIT-Fatigue (Cronbach alpha, 0.82-0.86) both had good internal consistency/reliability. At baseline, SF-36 and FACIT-Fatigue scores correlated significantly with Ankylosing Spondylitis Quality of Life scores (r = -0.36 to -0.66 and r = -0.70, respectively; all p < 0.0001). SF-36 scores varied by indicators of clinical severity, with greater impairment observed for more severe degrees of clinical activity (all p < 0.0001). FACIT-Fatigue scores correlated significantly with SF-36 scores (r = 0.42 to 0.74; all p < 0.0001) and varied by clinical severity (p < 0.05 to p < 0.0001).

**Conclusions:**

The SF-36 is a reliable, valid, and responsive measure of health-related quality of life and the FACIT-Fatigue is a brief and psychometrically sound measure of the effects of fatigue on patients with AS. These PROs may be useful in evaluating effectiveness of new treatments for AS.

**Trial Registration:**

ClinicalTrials.gov: NCT00085644 and NCT00195819

## Background

Ankylosing spondylitis (AS) is a chronic and progressive inflammatory disorder that primarily affects the axial skeleton, sacroiliac joints of the pelvis, and thoracic cage [1,2]. Patients experience pain, joint stiffness, and the eventual loss of spinal mobility with disease progression. Patients with AS frequently experience impaired physical function and well-being, require time away from work because of disability, and suffer from diminished health-related quality of life (HRQOL) [3-7]. The impact of AS on functioning and everyday life varies by patient, but most patients typically have a broad spectrum of impairments, including the physical, psychological, and social domains of HRQOL.

Patient-reported outcomes (PROs), including HRQOL assessments, symptom scales, and other measures, are increasingly used to evaluate the health-related outcomes of rheumatology treatments from the patient perspective. PROs are incorporated into clinical studies of patients with AS and provide important assessments of functioning and well-being that complement and expand on traditional clinical outcomes in AS [8]. AS impacts multiple HRQOL domains [6], including pain, physical function, fatigue, and psychological well-being [3,4,7-10]. Therefore, assessing HRQOL outcomes is important for a comprehensive understanding of the effectiveness of new treatments for AS. HRQOL outcome results from randomized controlled clinical trials permit physicians and patients to better understand and compare the health benefits of various therapies for AS.

Although disease-specific HRQL measures, such as the AS Quality of Life (ASQoL) Questionnaire, may be more sensitive to treatment effects and changes in clinical status, generic HRQL measures are useful for evaluating the burden of disease and for normative comparisons with general population samples. Generic HRQL measures, such as the Short Form 36 (SF-36) Health Survey, have been used to document the impact of chronic diseases on patient functioning and well-being, including AS [4]. Given the availability of general population samples, these generic HRQL measures can also be used for normative comparisons with chronic disease groups, such as AS, which can help interpret changes in HRQL related to treatment or disease progression. Fatigue is associated with rheumatoid arthritis and AS [3,4,6-10], and generic measures of fatigue may assist clinicians in understanding the experience of patients with AS.

Several PRO measures have been used in studies of patients with AS [8,11]. These measures include the SF-36 Health Survey [12,13]; the AS Quality of Life Questionnaire [14,15]; and the Revised Leeds Disability Questionnaire [16]. For application in clinical trials, it is necessary to demonstrate a measure's reliability, construct validity, and responsiveness [17,18]. The SF-36 has been used in several studies of patients with AS [12,19-22]. The developers of the ASQoL provided evidence supporting the reliability and validity of the measure [14,15]. However, additional information is needed to confirm the psychometric qualities of the SF-36 in AS.

The objective of the current analysis was to evaluate the psychometric characteristics of two PRO measures -- the SF-36 Health Survey and the Functional Assessment of Chronic Illness Therapy (FACIT)-Fatigue subscale -- in a sample of patients with AS. The psychometric characteristics examined included internal consistency/reliability, construct validity, and responsiveness to changes in patients' clinical disease activities. The current analyses are based on data from two completed clinical trials of adalimumab in patients with active AS [23,24]. Evidence supporting the psychometric characteristics of PROs is necessary for clinical trial analyses that compare treatments to support claims of HRQOL benefit [18].

## Methods

### Study design and patients

These psychometric analyses were completed based on clinical and PRO data collected from two Phase III, randomized, double-blind, placebo-controlled clinical trials that assessed the safety and clinical efficacy of subcutaneous injections of adalimumab in patients with AS. The two clinical trials -- the Adalimumab Trial Evaluating Long-Term Efficacy and Safety in Ankylosing Spondylitis (ATLAS) [23-25] and the M03-606 study [26] -- were similar in research design. ATLAS was completed in 43 centers in the United States and Europe (ClinicalTrials.gov identifier, NCT00085644), and the M03-606 study was conducted in 11 clinical centers in Canada (ClinicalTrials.gov identifier, NCT00195819).

Briefly, patients were 18 years of age or older with a diagnosis of AS according to the Modified New York criteria [27]. Patients also had to exhibit active disease, defined as meeting at least two of the following three conditions: Bath AS Disease Activity Index (BASDAI) score ≥4 (0-10-cm scale); total back pain score ≥4 on a 0-10-cm visual analog scale (VAS); or morning stiffness ≥1 hour. Inclusion criteria included an inadequate response to or intolerance of one or more nonsteroidal anti-inflammatory drugs or disease modifying antirheumatic drugs and a willingness to self-administer subcutaneous injections of adalimumab (HUMIRA^®^; Abbott Laboratories, Abbott Park, IL, USA) or matching placebo. Patients with radiologic evidence of total spinal ankylosis (bamboo spine) were excluded from participation in the M03-606 study, and enrollment of patients with total spinal ankylosis in ATLAS was limited to ≤10% of the total study sample.

In both studies, eligible patients were randomized to receive adalimumab 40 mg every other week or placebo for the initial 12-week, double-blind period of each study. The psychometric analyses described here were not designed to evaluate the treatment effects of adalimumab compared with placebo. This report describes only the methods and results of a blinded evaluation of the psychometric qualities of selected PROs assessed during the initial 12-week period of both clinical trials. Institutional review boards at participating clinical centers approved the protocol and all patients provided voluntary, written informed consent. Both studies were conducted in accordance with the Declaration of Helsinki.

### Patient-reported outcomes

Three PRO measures were included in this psychometric evaluation study: the SF-36 Health Survey, the FACIT-Fatigue scale, and the ASQoL. The SF-36 and ASQoL were included in both ATLAS and the M03-606 study, and were selected to comprehensively measure disease-specific (ASQoL) and generic (SF-36) domains of HRQOL. The FACIT-Fatigue was employed only in M03-606, and was selected to evaluate the impact of AS treatment on fatigue outcomes, as previous studies have demonstrated treatment effects on fatigue in patients with rheumatoid arthritis [28] and AS [23]. For the ASQoL and FACIT-Fatigue, data were collected at baseline, Week 2, and Week 12; the SF-36 was completed only at baseline and Week 12. The ASQoL was included to evaluate the construct validity of the other PRO measures.

#### Ankylosing Spondylitis Quality of Life Questionnaire

The ASQoL is a disease-specific instrument designed to measure quality of life in patients with AS and was developed using a needs-based model [15]. The instrument contains 18 yes or no items on the impact of AS "at this moment." The ASQoL has a total score ranging from 0 to 18, with lower scores representing better AS-specific quality of life. The instrument has good reliability and construct validity across several different AS populations [8,14,15,22].

#### Functional Assessment of Chronic Illness Therapy (FACIT)-Fatigue Subscale

The FACIT-Fatigue is a frequently used instrument that measures fatigue and its effect on functioning and daily activities [28]. The FACIT-Fatigue has 13 items answered on a 5-point rating scale based on a 7-day recall period. Scores range from 0 to 52, with lower scores reflecting greater fatigue. The instrument has good reliability and validity based on analyses of the general population in the United States, patients with cancer, and patients with rheumatoid arthritis [28-30], but a search of the medical literature indicated no published data on the psychometric qualities of the FACIT-Fatigue in AS patients.

#### Short Form 36 Health Survey

The SF-36 Health Survey is a generic health status instrument developed for application in primary care and chronic disease populations [13]. The SF-36 Version 1, with a 4-week recall period, was used in this study. The SF-36 contains two summary scores (Mental Component Summary [MCS] and Physical Component Summary [PCS] scores), and the following eight subscales: physical function, bodily pain, role-physical, general health, vitality, social function, role-emotional, and mental health. The SF-36 subscales and summary scores have excellent reliability and good construct validity across the general US population and chronic disease populations [13,31], including in patients with AS [4,19-22,32,33].

#### Bath Ankylosing Spondylitis Disease Activity

The BASDAI is a six-item measure of disease activity and includes questions on fatigue, spinal pain, peripheral arthritis, enthesitis (i.e., inflammation at the attachment of ligaments or tendons to bone), and morning stiffness [34]. The BASDAI is a well-established instrument widely used in clinical studies to evaluate AS disease activity. All items are patient reported using a 0-10 VAS, and lower scores indicate less disease activity.

#### Bath Ankylosing Spondylitis Functional Index

The BASFI consists of 10 questions related to daily activities. The original BASFI used a 0 to 10 VAS for each of the 10 questions, for which 0 indicates that an activity was performed without difficulty and 10 indicates that an activity was impossible to perform. The mean of these yields the final BASFI score of 0-10 [35]. However, in ATLAS and the M03-606 study, patients answered each of the same 10 questions using a 0-100-mm VAS, and the mean gave a final BASFI score of 0-100.

### Clinical measures

The Assessment of SpondyloArthritis international Society (ASAS) [36] was used in these psychometric analyses. The ASAS response criterion [36] consists of the percentage of improvement in three of the following four domains: patient's global assessment of disease activity VAS, pain, function (represented as the mean BASFI score [35]), and inflammation (represented as the mean of the two morning stiffness-related BASDAI questions). The ASAS 20% improvement (ASAS20), ASAS50, and ASAS70 response criteria were used in the clinical trials and have been applied by regulatory agencies to evaluate the clinical efficacy of tumor necrosis factor (TNF) antagonists for the treatment of AS. For the psychometric analyses based on ASAS response criteria, patients were categorized into mutually exclusive groups corresponding to at least ASAS20, at least ASAS50, or at least ASAS70 response criteria based on the Week 12 assessment.

### Statistical analyses

The psychometric qualities of the PROs were assessed to determine reliability, validity, and responsiveness [17,37]. The internal consistency/reliability of the multi-item instruments and subscales was evaluated using the Cronbach alpha coefficient [38]. Reliabilities were estimated at baseline and at Week 12. A Cronbach alpha coefficient of > 0.70 was indicative of acceptable internal consistency/reliability of group comparisons [17]. We examined the item-total correlations (corrected for overlap) as another indicator of reliability for the SF-36 subscales and FACIT-Fatigue. In addition, we replicated the factor analysis of the SF-36 subscales to verify the factor structure underlying the PCS and MCS scores.

Validity reflects the extent to which an instrument or subscale actually measures the construct it is intended to measure [17]. Validity of the PROs was examined by specifying and testing hypotheses about the relationships between the measures and clinical assessments or other PRO measures. We evaluated the relationship between SF-36 scores and FACIT-Fatigue scores and selected clinical measures (specifically the BASDAI total score, the BASDAI fatigue question, the BASDAI pain question, total back pain, BASFI, patient's global assessment of disease activity, and the physician's global assessment of disease activity). We hypothesized that the BASDAI, BASFI, BASDAI fatigue question, and patient global assessment of disease activity would have moderate to strong (0.40-0.60) correlations with the FACIT-Fatigue, and SF-36 PCS scores, while the pain and physician global measures would have moderate to strong correlations with the SF-36 PCS, and low to moderate (0.20-0.40) correlations with FACIT-Fatigue. We consider correlations < 0.30 to be low; correlations 0.30 to 0.60 to be moderate; and correlations > 0.60 to be strong. In addition, we examined the relationships between the FACIT-Fatigue and SF-36 subscales and summary scores and the ASQoL scores. Pearson's correlation coefficients were used to evaluate the strength and direction of these associations at baseline and at Week 12.

Analysis of covariance (ANCOVA) models, with adjustment for age and sex, were used to evaluate mean PRO scores by clinical severity based on total BASDAI score (≥7, 4-6.9, or < 4), BASDAI fatigue score (≥7, 4-6.9, or < 4), BASDAI pain score (≥7, 4-6.9, or < 4), pain score (≥66, 34-65, or < 34), total back pain score (≥66, 34-65, or < 34), BASFI score (≥66, 34-65, or < 34), and patient's and physician's global assessments of disease activity (≥66 vs. < 66). These categories were defined through review of sample distributions and designed to divide patients roughly into groups representing severe, moderate, and mild disease.

The responsiveness of the FACIT-Fatigue and SF-36 subscales and summary scores was evaluated by determining the association between the ASAS and the PRO measures. ANCOVA models were used to estimate least-square mean baseline to 12-week change scores for the SF-36 subscales, as well as the PCS, MCS, and FACIT-Fatigue scores. The ANCOVA models included factors for the ASAS response group (i.e. < 20%; ≥20% to < 50%; ≥50% to < 70%; and ≥70%), age, sex, and the relevant baseline PRO score. The clinical responsiveness analyses focused on the baseline to 12-week changes in the clinical and PRO measures. Effect-size estimates were also included for interpretation purposes.

All statistical tests were based on an alpha of 0.05, with no adjustments for multiple statistical tests. The results were interpreted with consideration for the number of statistical analyses performed.

## Results

ATLAS enrolled 315 patients and the M03-606 study enrolled 82 patients. The average age of patients with AS who participated in the two clinical trials was 42.0 years (SD, 11.5), and the sample was mostly male (75.8%) and white (95.7%) (Table 1). At baseline, the mean BASFI score was 53.9 (SD, 21.9), the mean BASDAI score was 6.3 (SD, 1.7), and the mean ASQoL score was 10.5 (SD, 4.3). The average duration of AS was 11.3 years (SD, 9.4).

**Table 1:** Baseline demographic and clinical characteristics (N = 397)

Characteristic^a^	
Age, years	42.0 (11.5)

Sex, n (%)	

Female	96 (24.2)

Male	301 (75.8)

Race, n (%)	

White	380 (95.7)

Non-white	17 (4.3)

HLA-B27, n (%)	

Negative	74 (18.9)

Positive	317 (81.1)

Duration of AS, years	11.3 (9.4)

Patient's global assessment of disease activity, 0-100-mm VAS	64.2 (20.7)

BASFI, 0-100 VAS	53.9 (21.9)

Inflammation, 0-100-mm VAS	67.7 (20.2)

Total back pain, 0-100-mm VAS	66.2 (20.2)

BASDAI, 0-10-cm VAS	6.3 (1.7)

C-reactive protein, mg/dL	1.9 (2.4)

^a ^Data are mean (SD) unless otherwise noted.

### Patient-reported outcome descriptive statistics and reliability

Complete baseline and Week 12 data were available for 98.2% of patients in the two clinical trials. The baseline means, standard deviations, and internal consistency/reliability coefficients for the FACIT-Fatigue and SF-36 subscale scores are summarized in Table 2. The internal consistency/reliability coefficients for the FACIT-Fatigue were 0.82 at baseline and 0.86 at Week 12. All reliability coefficients for the SF-36 subscales exceeded 0.75 at both baseline and Week 12, except for general health at baseline (0.74) (Table 2). For the FACIT-Fatigue, item-total correlations were 0.56-0.88 for both visits. At baseline, item-total correlations for the SF-36 subscales were 0.35-0.74 for physical function; 0.49-0.61 for role-physical; both 0.62 for bodily pain; 0.31-0.68 for general health; 0.57-0.64 for vitality; both 0.68 for social function; 0.59-0.72 for role-emotional; and 0.55-0.77 for mental health. The item-total correlations for the SF-36 domain scores were comparable at 12 weeks (data not shown). We replicated the factor analysis of the SF-36 subscale scores and found a comparable factor structure to that reported by Ware et al. [13] (data not shown).

**Table 2:** Baseline descriptive statistics and internal consistency reliability coefficients for patient-reported outcomes

Patient-reported outcome measure	N	Mean (SD)	Internal consistency reliability^a^	
			**Baseline**	**Week 12**

FACIT-Fatigue	82	24.0 (10.2)	0.82	0.86

SF-36				

Physical function	396	47.8 (22.0)	0.87	0.92

Role-physical	397	19.3 (29.3)	0.76	0.84

Bodily pain	397	31.0 (15.8)	0.77	0.89

General health	396	42.2 (20.0)	0.74	0.78

Vitality	397	33.0 (17.6)	0.78	0.88

Social function	397	54.9 (24.2)	0.80	0.84

Role-emotional	395	52.2 (42.6)	0.82	0.83

Mental health	397	62.2 (18.7)	0.83	0.87

^a ^Cronbach alpha.

### Relationships between patient-reported outcomes

The correlations between the ASQoL and the FACIT-Fatigue were -0.70 (p < 0.0001) at baseline and -0.81 (p < 0.0001) at Week 12. Correlations between the ASQoL, FACIT-Fatigue, and SF-36 subscales and summary scores are reported in Table 3. ASQoL scores were significantly correlated with SF-36 summary scores at both baseline and Week 12 (p < 0.0001), with the greatest baseline correlations between the ASQoL and social function (r = -0.66, p < 0.0001), bodily pain (r = -0.60, p < 0.0001), and physical function (*r *= -0.59, p < 0.0001). The Week 12 correlations were greater than the baseline correlations (Table 3).

**Table 3:** Relationship between ASQOL, FACIT-Fatigue, and SF-36 subscales and summary scores^a^

SF-36 scores	Baseline		Week 12	
	**ASQoL**	**FACIT-F**	**ASQoL**	**FACIT-F**

Physical function	-0.59	0.58	-0.73	0.64

Role-physical	-0.50	0.53	-0.73	0.65

Bodily pain	-0.60	0.56	-0.76	0.75

General health	-0.49	0.50	-0.65	0.57

Vitality	-0.53	0.74	-0.72	0.82

Social function	-0.66	0.67	-0.77	0.73

Role-emotional	-0.39	0.42	-0.64	0.61

Mental health	-0.53	0.42	-0.67	0.64

Physical Component Summary (PCS)	-0.50	0.54	-0.70	0.63

Mental Component Summary (MCS)	-0.51	0.53	-0.65	0.71

^a ^p < 0.0001 for all correlations.

FACIT-Fatigue scale scores were significantly correlated with SF-36 subscale scores at baseline and at 12 weeks (Table 3). The greatest correlations were between the FACIT-Fatigue score and the SF-36 vitality subscale score (*r *= 0.74 at baseline and 0.82 at Week 12, both p < 0.0001). FACIT-Fatigue scores were also well-correlated at baseline with social function (*r *= 0.67, p < 0.0001), physical function (*r *= 0.58, p < 0.0001), and bodily pain (*r *= 0.56, p < 0.0001). The correlations for the Week 12 scores were similar but greater. The PCS and MCS scores were both significantly correlated with FACIT-Fatigue scores at baseline (*r *= 0.54 and *r *= 0.53, respectively, both p < 0.0001) and Week 12 (r = 0.63 and r = 0.71, respectively, both p < 0.0001).

### Relationships between patient-reported outcomes and clinical measures

FACIT-Fatigue scores were correlated with all of the selected clinical outcome measures (Table 4). The FACIT-Fatigue was most substantially correlated with the BASDAI fatigue item (*r *= -0.69, *P *< 0.0001), the BASDAI (*r *= -0.60, p < 0.0001), and the BASFI (*r *= -0.56, p < 0.0001). For the SF-36, the greatest correlations were observed between the clinical assessments and the physical function (*r *= -0.36 to *r *= -0.72, all p < 0.0001) and bodily pain subscale scores (*r *= -0.42 to *r *= -0.64, all p < 0.0001). In general, the PCS was more substantially correlated with the clinical outcome measures than the MCS (Table 4). The BASDAI was significantly correlated with the PCS (*r *= -0.47, p < 0.0001) and MCS (r = -0.22, p < 0.0001). The BASFI was more strongly correlated with the PCS (*r *= -0.65, p < 0.0001) than the MCS (*r *= -0.15, p < 0.05).

**Table 4:** Relationship between patient-reported outcome and clinical measures^a^

Patient-reported outcome measure	Clinical outcome measures						
	**BASDAI**	**BASDAI fatigue**	**BASDAI pain**	**Total back pain**	**BASFI**	**Patient's global assessment of disease severity**	**Physician's global assessment of disease severity**

FACIT-Fatigue	-0.60	-0.69	-0.47	-0.27	-0.56	-0.41	-0.25

SF-36							

Physical function	-0.45	-0.36	-0.40	-0.41	-0.72	-0.46	-0.36

Role-physical	-0.37	-0.32	-0.29	-0.37	-0.40	-0.42	-0.29

Bodily pain	-0.58	-0.45	-0.55	-0.55	-0.57	-0.64	-0.42

General health	-0.31	-0.30	-0.25	-0.20	-0.38	-0.30	-0.31

Vitality	-0.35	-0.49	-0.21	-0.24	-0.33	-0.30	-0.10^a^

Social function	-0.38	-0.41	-0.25	-0.30	-0.42	-0.37	-0.24

Role-emotional	-0.22	-0.22	-0.16^a^	-0.16^a^	-0.19^a^	-0.20	-0.18^a^

Mental health	-0.24	-0.27	-0.15^a^	-0.12^a^	-0.21	-0.18^a^	-0.10^a^

PCS	-0.47	-0.36	-0.43	-0.46	-0.65	-0.52	-0.40

MCS	-0.22	-0.30	-0.11^a^	-0.11^a^	-0.15^a^	-0.16^a^	-0.10^a^

^a ^p < 0.001 for correlations indicated.

**Figure 1:**
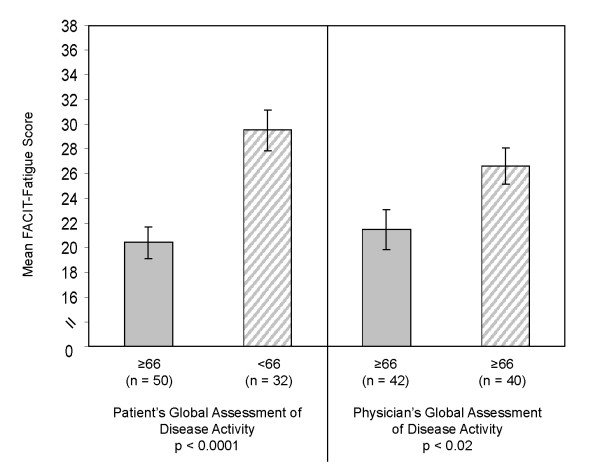
**FACIT-Fatigue Scores by Disease Activity**. Mean (SE) Functional Assessment of Chronic Illness Therapy (FACIT)-Fatigue subscale scores by patient's and physician's global assessment of disease activity.

ANCOVA models, adjusting for age and sex, were used to evaluate the association between clinical severity and the PRO measures (Table 5). For the FACIT-Fatigue, there were statistically significant differences in mean scores by BASDAI (p < 0.0001), BASDAI fatigue (p < 0.0001), BASDAI pain (p = 0.0002), total back pain (p = 0.013), and BASFI (p < 0.0001). As clinical severity increased, mean FACIT-Fatigue scores decreased (i.e. worse fatigue symptoms). For example, for the BASDAI fatigue item, mean FACIT-Fatigue scores were least for those patients in the most severe group compared with the less severe groups (Table 5). Similar patterns of mean FACIT-Fatigue scores were observed for the other clinical severity measures. Patients who rated their disease activities ≥66 had mean FACIT-Fatigue scores of 20.4 compared with those who rated their disease activities <66 (mean FACIT-Fatigue, 29.5) (p < 0.0001, Figure 1).

**Table 5:** Relationship between clinical severity and selected patient-reported outcomes

Clinical Severity Measure	Patient-reported outcome measure		
	**FACIT-Fatigue Mean (SE)**	**PCS** **Mean (SE)**	**MCS** **Mean (SE)**

**BASDAI, 0-10 VAS**			

≥7	17.55 (1.64)	29.25 (0.57)	40.23 (1.01)

4 to 6.9	26.08 (1.26)	33.22 (0.50)	45.19 (0.69)

<4	37.86 (2.32)	40.77 (1.25)	45.29 (1.88)

p-value	< 0.0001	< 0.0001	= 0.0003

**BASDAI fatigue, 0-10 VAS**			

≥7	18.50 (1.30)	30.27 (0.49)	40.20 (0.78)

4 to 6.9	27.24 (1.58)	34.09 (0.65)	45.95 (0.84)

< 4	36.18 (1.24)	37.95 (1.24)	48.94 (1.70)

p-value	< 0.0001	< 0.0001	< 0.0001

**BASDAI pain, 0-10 VAS**			

≥7	20.47 (1.39)	30.20 (0.45)	42.56 (0.76)

4 to 6.9	29.14 (1.64)	35.71 (0.68)	45.41 (0.88)

< 4	29.25 (4.50)	39.66 (1.65)	41.23 (2.20)

p-value	= 0.0002	< 0.0001	= 0.298

**Total back pain, 0-100 VAS**			

≥66	21.60 (1.46)	30.00 (0.47)	42.71 (0.78)

34 to 65	27.99 (1.65)	35.29 (0.65)	43.78 (0.91)

< 34	24.00 (8.00)	38.98 (1.33)	46.60 (1.90)

p-value	= 0.013	< 0.0001	= 0.076

BASFI, 0-100 VAS			

≥66	17.63 (1.60)	26.83 (0.47)	41.57 (1.10)

34 to 65	24.88 (1.45)	33.45 (0.48)	44.06 (0.79)

< 34	33.62 (2.19)	39.51 (1.78)	44.77 (1.14)

p-value	< 0.0001	< 0.0001	= 0.028

**Figure 2:**
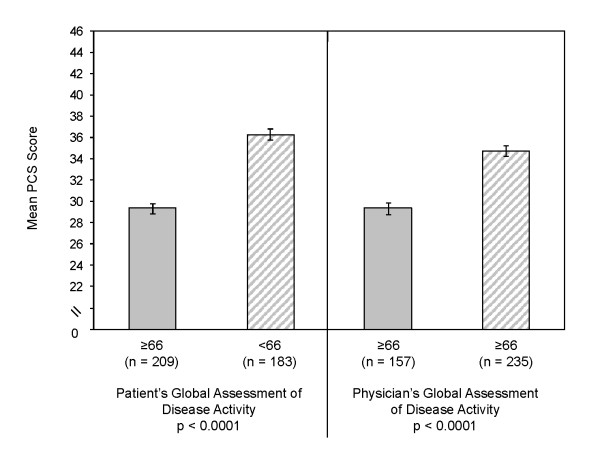
**SF-36 PCS Scores by Disease Activity**. Mean (SE) SF-36 Physical Component Summary (PCS) scores by patient's and physician's global assessments of disease activity.

We also compared mean PCS and MCS scores by the clinical severity measures (Table 5). For the PCS, there were statistically significant differences in mean scores by BASDAI, BASDAI fatigue, BASDAI pain, total back pain, and BASFI (all p < 0.0001). In all cases, mean PCS scores were worse (i.e. lower indicating impaired physical health status) for those patients reporting greater clinical severities. Mean PCS scores varied significantly by patient's and physician's global assessments of disease severity (p < 0.0001, Figure 2). For the MCS, there were statistically significant differences in mean scores by BASDAI (p = 0.0003), BASDAI fatigue (p < 0.0001), and BASFI (p = 0.028), but not for BASDAI pain (p = 0.289), total back pain (p = 0.076), or the physician's global assessment (p = 0.750). The mean MCS scores were generally better for patients reporting lower clinical severity of symptoms. For example, for BASDAI fatigue, MCS scores were most impaired for the most severe group (mean BASDAI fatigue, 40.2), less impaired for the moderate group (mean BASDAI fatigue, 45.9), and best for the mild group (mean BASDAI fatigue, 48.9). Mean MCS scores varied significantly by patient's global ratings (p = 0.021) but not the physician's global assessment of disease (data not shown).

### Responsiveness of patient-reported outcomes

Clinical responsiveness was evaluated by determining the relationships between mean baseline to Week 12 changes in FACIT-Fatigue and SF-36 scores by ASAS response criteria (i.e. non-responders and 20%, 50%, or 70% responders). The ASAS responder groups achieved statistically significant improvements in FACIT-Fatigue scores compared with the nonresponder group (p < 0.0001). Differences in mean baseline to 12-week change scores between the nonresponder group and the responder groups were 9.7 points for ASAS20, 14.6 points for ASAS50, and 15.4 points for ASAS70 (Table 6). FACIT-Fatigue change scores for patients meeting ASAS70 response criteria were fairly similar to those meeting ASAS50 response criteria.

**Table 6:** Baseline to Week 12 mean changes in patient-reported outcomes according to ASAS working group response criteria

Patient-reported outcome measure	ASAS Response	ASAS Response	ASAS Response	ASAS Response	p-value
	**Nonresponder**	**ASAS20**	**ASAS50**	**ASAS70**	

**Mean change from baseline to Week 12 (SE)** **Effect Size (ES)**					

FACIT-Fatigue	-0.24 (0.95)	9.42 (1.24)	14.33 (1.89)	15.20 (2.50)	< 0.0001

FACIT-Fatigue	ES = 0.02	ES = 0.92	ES = 1.40	ES = 1.49	< 0.0001

FACIT-Fatigue	n = 54	n = 12	n = 6	n = 10	< 0.0001

SF-36					

PCS	0.79 (0.42)	6.36 (1.00)	11.38 (1.12)	14.28 (0.77)	<0.0001

PCS	ES = 0.10	ES = 0.81	ES = 1.44	ES = 1.81	< 0.0001

PCS	n = 217	n = 63	n = 42	n = 60	< 0.0001

MCS	0.54 (0.70)	3.33 (1.24)	1.86 (1.70)	9.61 (1.14)	< 0.0001

MCS	ES = 0.05	ES = 0.30	ES = 0.17	ES = 0.86	< 0.0001

MCS	n = 217	n = 63	n = 42	n = 60	< 0.0001

Physical function	0.48 (1.11)	14.82 (1.98)	17.30 (2.90)	25.34 (2.72)	< 0.0001

Physical function	ES = 0.02	ES = 0.67	ES = 0.79	ES = 1.15	< 0.0001

Physical function	n = 222	n = 64	n = 43	n = 60	< 0.0001

Role- physical	5.31 (1.80)	22.66 (4.16)	37.21 (6.30)	55.00 (4.40)	< 0.0001

Role- physical	ES = 0.18	ES = 0.77	ES = 1.27	ES = 1.88	< 0.0001

Role- physical	n = 223	n = 64	n = 43	n = 60	< 0.0001

Bodily pain	3.03 (0.96)	17.50 (2.18)	30.40 (2.97)	41.02 (2.24)	< 0.0001

Bodily pain	ES = 0.19	ES = 1.11	ES = 1.92	ES = 2.60	< 0.0001

Bodily pain	n = 223	n = 64	n = 43	n = 60	< 0.0001

General health	-0.12 (0.93)	4.78 (1.84)	13.19 (2.50)	21.40 (2.17)	< 0.0001

General health	ES = 0.01	ES = 0.24	ES = 0.66	ES = 1.07	< 0.0001

General health	n = 221	n = 63	n = 42	n = 60	< 0.0001

Vitality	2.50 (1.07)	13.52 (1.84)	18.49 (2.68)	31.56 (2.36)	< 0.0001

Vitality	ES = 0.14	ES = 0.77	ES = 1.05	ES = 1.79	< 0.0001

Vitality	n = 222	n = 64	n = 43	n = 60	< 0.0001

Social function	1.79 (1.36)	11.91 (2.62)	9.01 (3.36)	27.08 (2.63)	< 0.0001

Social function	ES = 0.07	ES = 0.49	ES = 0.37	ES = 1.12	< 0.0001

Social function	n = 223	n = 64	n = 43	n = 60	< 0.0001

Role- emotional	3.32 (2.88)	13.54 (5.74)	15.50 (7.87)	32.22 (4.95)	< 0.0001

Role- emotional	ES = 0.08	ES = 0.32	ES = 0.36	ES = 0.76	< 0.0001

Role- emotional	n = 221	n = 64	n = 43	n = 60	< 0.0001

Mental health	-0.01 (1.09)	5.98 (1.63)	4.23 (2.54)	17.20 (1.77)	< 0.0001

Mental health	ES = 0.00	ES = 0.32	ES = 0.23	ES = 0.92	< 0.0001

Mental health	n = 222	n = 64	n = 43	n = 60	< 0.0001

There were statistically significant differences in mean baseline to 12-week changes in PCS scores between the ASAS responder groups (p < 0.0001, Table 6). After 12 weeks of treatment, mean change scores from baseline for SF-36 PCS scores were significantly greater for patients who responded to therapy compared with those who did not respond to therapy (p < 0.001). Changes in SF-36 PCS scores were lowest for ASAS nonresponders and greatest for ASAS70 responders. Mean changes for the ASAS50 responders were almost double the changes for the ASAS20 responders, and mean changes in the ASAS70 responders were more than double the changes in the ASAS20 responders.

There were also statistically significant differences in mean baseline to Week 12 MCS scores across different ASAS responder groups (p < 0.0001, Table 6). The baseline to 12-week change scores for patients achieving the ASAS20 and ASAS70 response criteria were 2.8 and 9.1 points greater, respectively, compared with non-responders (p < 0.0001). The ASAS50 responders actually had mean change scores that were less than those of ASAS20 responders, but greater than those of nonresponders.

We compared the mean baseline to Week 12 changes in the SF-36 subscale scores by ASAS responder status (Table 6). There were statistically significant improvements in all of the SF-36 subscale scores between non-responder and responder groups (all p < 0.0001). For example, for SF-36 physical function scores, we observed a 14.3-point improvement in the ASAS20 responder group, a 16.8-point improvement in the ASAS50 responder group, and a 24.9-point improvement in the ASAS70 responder group, all compared with the non-responder group. These differences in mean change scores were seen across most of the SF-36 subscales, except for mental health and social function, with the greatest effects observed in the ASAS50 and ASAS70 responder groups.

## Discussion

Patient-reported outcomes, such as HRQOL, functional status, and fatigue measures, are increasingly used to examine the effectiveness of new therapies for AS [19,23,39-42], but there is little documentation as to the reliability, validity, and responsiveness of these measures in patients with AS. We evaluated the psychometric characteristics of two instruments -- the SF-36 Health Survey and FACIT-Fatigue -- based on a secondary analysis of blinded clinical trial data from a large sample of patients with AS. The results indicate that the SF-36 is a reliable and valid measure of HRQOL in patients with AS. Although based on a smaller sample size, there is good evidence supporting the psychometric qualities of the FACIT-Fatigue subscale in AS patient populations.

The ASQoL is a disease-specific measure of quality of life with evidence supporting reliability and validity [14,15]. However, recent qualitative research suggests that the ASQoL may not cover all important and frequently mentioned patient concerns about HRQOL [43]. We believe that the addition of the SF-36 and FACIT-Fatigue scales helps provide a more comprehensive assessment of the main health outcomes important to patients with AS.

The SF-36 summary scores and subscales (or domains) were also found to have acceptable reliability and good evidence of validity in this sample of patients with AS. In addition, we found a comparable factor solution for the PCS and MCS using the AS sample. Significant relationships between the SF-36 scores and ASQoL, FACIT-Fatigue, and clinical endpoints were observed. We observed an increase in the correlations among the patient-reported outcomes at the 12-week assessment, and this increase was likely attributable to the more restrictive ranges in patient-reported outcome scores at baseline because of clinical trial entry criteria. Restricted ranges in scores may attenuate the correlation coefficients.

The SF-36 scores, especially those measuring physical function and pain, were responsive to clinical improvements as assessed with the ASAS response criteria. For example, there was a 5.6-point (SD = 6.6) difference in PCS scores between ASAS nonresponders and ASAS20 responders, a 10.6-point (SD = 6.2) difference between nonresponders and ASAS50 responders, and a 13.5-point (SD = 6.1) difference between nonresponders and ASAS70 responders. Less consistent findings were observed for the MCS. ASAS50 and ASAS70 responder groups achieved greater improvements vs. the non-responders or ASAS20 responders for measures of physical and role function, pain, general health, and vitality. Published clinical trials in AS have found that several SF-36 subscale scores are responsive to treatment effects [11,19,21,39-42]. Certainly, 5-point differences or mean changes in PCS or MCS scores are clinically relevant, and lesser changes of 2.5 to 3.0 points are likely to be clinically meaningful.

The AS patients in the current analysis had significant impairment in health status at baseline, consistent with previous studies [4,32]. In the current analysis, mean baseline PCS and MCS scores were 32.6 and 43.4, respectively. The mean scores for these patients with AS are considerably lower than mean scores of the general US population [29], with differences of 1.7 standard deviation units for the PCS and 0.7 standard deviation units for the MCS. The SF-36 subscales scores for the current analysis are also less than those reported by Dagfinrud and colleagues [4] and Chorus and colleagues [32]. Therefore, the current analysis provides additional evidence of the significant impairment in health status and functioning in AS across multiple domains of physical and role functions, pain, energy, emotional well-being, and general health perceptions.

FACIT-Fatigue was also shown to have good reliability and validity in this sample of patients with AS. This measure focuses on fatigue-related problems and concerns and was originally developed to assess fatigue in oncology patients [29,30,44] but has been applied to other chronic diseases, such as rheumatoid arthritis [28,45]. In this AS sample, we found reliabilities exceeding 0.80 and statistically significant relationships between the FACIT-Fatigue and measures of vitality, physical function, role-physical, social function, and clinical severity.

As expected, the FACIT-Fatigue scores were most closely related to similar endpoints, such as the SF-36 vitality score and the BASDAI fatigue item. However, meaningful associations were observed for the other patient-reported and clinical outcomes, supporting the validity of the FACIT-Fatigue. These findings for AS patients support the psychometric qualities of the FACIT-Fatigue for application in clinical studies of other populations with rheumatic diseases, such as patients with rheumatoid arthritis [28,45]. In fact, the mean baseline FACIT-Fatigue scores observed in these patients with AS were much less than those observed for patients with rheumatoid arthritis (mean, 23.9 vs. 29.2) or the general US population [29]. These results suggest that fatigue should be a focus of attention in the treatment of AS.

FACIT-Fatigue was responsive to changes in clinical status based on the ASAS response criteria. We observed significant improvements in FACIT-Fatigue scores, with the greatest mean changes observed for patients meeting ASAS50 or ASAS70 response criteria. FACIT-Fatigue scores demonstrated a 9.7-point difference in improvement between nonresponders and ASAS20 responders. These differences significantly exceeded the minimum clinically important difference of 3 to 4 points validated for patients with rheumatoid arthritis [28]. For AS, differences of 4 to 5 points in FACIT-Fatigue scores may be clinically meaningful; however, further confirmation is needed.

The results of these psychometric analyses are limited to patients participating in one of these two clinical trials, and may not be generalizable to all patients with AS. However, we propose that, based on the strengths of the secondary analysis, including comparative clinical measures, a well-defined patient population, and longitudinal data all provide good evidence supporting the psychometric characteristics of the SF-36 and FACIT-Fatigue for AS patients.

## Conclusions

In summary, our analysis provides additional evidence supporting the reliability and validity of the SF-36 and FACIT-Fatigue in patients with AS. The SF-36 has been widely used in rheumatoid arthritis and AS clinical trials, and this analysis demonstrated that this generic health status measure is psychometrically sound and responsive in AS. As AS has broad and extensive impacts on HRQOL, comprehensive measures of patient outcomes are necessary for evaluating the effectiveness of new treatments. The FACIT-Fatigue has not been widely used in AS studies. However, we have provided evidence supporting its validity and, more importantly, its responsiveness. Based on these findings, a PRO battery consisting of the ASQoL, SF-36, and FACIT-Fatigue scale represents a useful, valid, and responsive approach to fully capturing effects of treatment on the health outcomes of AS patients. These PRO data, combined with clinical endpoints, may also assist physicians and their patients in determining the most effective treatments for AS.

## Abbreviations

ANCOVA: analysis of covariance; AS: ankylosing spondylitis; ASAS: Assessment of SpondyloArthritis international Society; ASQoL: AS Quality of Life; ATLAS: Adalimumab Trial Evaluating Long-Term Efficacy and Safety in Ankylosing Spondylitis; BASDAI: Bath AS Disease Activity Index; FACIT: Functional Assessment of Chronic Illness Therapy; HRQOL: health-related quality of life; MCS: Mental Component Summary score; PCS: Physical Component Summary score; PROs: patient-reported outcomes; SF-36: Short Form 36 Health Survey; TNF: tumor necrosis factor; VAS: visual analog scale.

## Competing interests

Anne Rentz and Dennis Revicki received research support from Abbott Laboratories for the analyses and preparation of the manuscript. Robert Wong and Michelle Luo were employees at Abbott Laboratories when this research was conducted.

## Authors' contributions

AMR, DAR, MPL, and RLW developed the design of the statistical analysis plan for this psychometric analysis. DAR and AMR executed the analysis, and AMR, DAR, MPL, and RLW interpreted the results of the analysis and prepared and revised the manuscript. All authors read and approved the final manuscript.
